# E-commerce channel management on the manufacturers’ side: ongoing debates and future research pathways

**DOI:** 10.1007/s11846-023-00645-w

**Published:** 2023-03-21

**Authors:** Jacopo Ballerini, Dorra Yahiaoui, Guido Giovando, Alberto Ferraris

**Affiliations:** 1 University of Turin, Turin, Italy; 2 Vrije Universiteit, Amsterdam, The Netherlands; 3 Kedge Business School, Marseille, France; 4 Laboratory for International and Regional Economics, Graduate School of Economics and Management, Ural Federal University, Yekaterinburg, Russia; 5 European Centre for Business Research, Pan-European University, Bratislava, Czech Republic

**Keywords:** Online channel, E-commerce, Channel management, Bibliographic coupling, Literature review, L20, L22, L25, M10, M15, M30

## Abstract

Since the beginning of the 2000s, online commerce has been gradually taking over and shaping the global marketplace. This has led several scholars to study the phenomenon from different angles, from consumer habits to privacy risks to related technological innovations. However, only recently has a branch of literature addressing the online channel management phenomenon from the manufacturers’ perspective emerged. This rapidly expanding literature strand remains rather fragmented, raising the need for a systematic literature review to comprehensively structure and discuss it. This study, accordingly, proposes a systematic literature review on online channel management from the manufacturers’ perspective. Firstly, it provides relevant bibliometric insights into the ongoing research on the topic. Secondly, applying the bibliographic coupling methodology individuates 92 interconnected contributions published by 31 December 2021. Three different, albeit interconnected, thematic clusters are discovered and reviewed, revealing their focus on (a) strategic marketing issues around manufacturervsretailer conflict, (b) pricing policies and trade-offs among pricevsservices, and (c) operational interactions and strategies between supply chain members. Finally, after a systematic literature review the authors develop thirteen original research propositions concerning new research pathways and theoretical advancements to be designed and implemented.

## Introduction

The digital revolution of the first two decades of the new millennium have brought with them numerous changes in product sales channels and, as a result, in consumer habits and expectations (Bresciani et al. 2022; Weyer et al. 2020). One of the most impactful phenomena from a global retail perspective is undoubtedly e-commerce. It is significant to think that a company like Amazon, already a billionaire leader in the online channel, increased its revenues more than tenfold between 2010 and 2020, from 34 to 389 billion dollars (Coppola 2021). In addition, the COVID-19 pandemic crisis has further irrevocably shaped consumer habits, who increasingly prefer to make purchases online (Vázquez-Martínez et al. 2021). The online channel is thus progressively destined to acquire greater relevance in the total market, shaping the entire entrepreneurial ecosystem (Song et al. 2022). Undoubtedly, digital entrepreneurs have the opportunity to create new digitally native business models adapted to consumers’ changing needs (Kraus et al. 2018). Less immediate, however, is the adaptation to this rising distribution channel that traditional manufacturing companies, comprising the major portion of the global industrial fabric, must undertake. Therefore, scholars are expected to pay special attention to this business category and its online selling opportunity.

There are several ways in which companies and different stakeholders can engage in e-commerce, which affects numerous companies’ functional aspects. Unsurprisingly, research on the topic has flourished, as witnessed by the numerous literature reviews that address the subject from different perspectives. Other literature reviews have focused on consumers’ online shopping habits. For instance, Mishra et al. (2021) reviewed the studies that addressed consumers’ cognitive decision-making when confronted with omnichannel shopping, or, with a more technical slant, Alamdari et al. (2020) systematically reviewed the literature investigating online recommendation system tools that increase shoppers’ user experience. Moreover, the literature review on Twitter adoption by Cano-Marin et al. (2023) ascribes social media a role in forecasting online sales. Other scholars have produced literature reviews from a purely logistical point of view. Indeed, Mangiaracina et al. (2019) comprehensively address the literature focusing on the B2C last mile delivery solutions, while other authors have provided reviews of the literature addressing the e-fulfillment issues encountered by omni-channel retailers (Melacini et al. 2018). Other researchers have produced literature reviews looking at the factors affecting the sutainability level of e-commerce packaging (Escursell et al. 2021). Finally, another strand of scholars has provided literature reviews from an IT security angle. For instance, bt Mohd and Zaaba (2019) reviewed all the criteria provided by the literature to evaluate e-commerce websites’ degree of security.

A significant number of scholars recently turned their attentions towards adopting the resource-based view (RBV) and capabilities perspective to identify optimal organizational and individual capabilities in enhancing business e-commerce performance, be it new digital business models, service companies, or distributors of physical products (Cui and Pan 2015; Fuller et al. 2022; Gregory et al. 2019; Saini and Johnson 2005).

Alongside them, there is also a productive niche of researchers who have focused on the peculiarities of online channel management from the perspective of manufacturing companies identifying the multiple selling formats that manufacturers can choose to adopt. Firstly, manufacturers can sell directly through a proprietary website like Nike, HP, Apple, Mattel, etc., or sometimes they opt explicitly not to take action on the online channel like Levi’s in order to avoid the risk of channel competition, price erosion, or cannibalization (Kumar and Ruan 2006; Matsui 2016). A second approach represents the involvement of specialized third-party online platforms, acting as a marketplace through which manufacturers sell their goods by paying a fee for the service like Microsoft or Samsung do on Bestbuy (Hagiu and Wright 2015; Qi et al. 2020), identified as agency selling. Finally, manufacturers can opt to distribute their goods to specialized (or non-specialized) online players, like Crocs, Inc. does with Amazon or Zappos, which resell to final consumers and contain standard B2B dynamics (Pu et al. 2020). However, the most recent scientific contributions are still fragmented into different streams approaching the subject from different viewpoints (Pu et al. 2020). In addition, no comprehensive literature review specifically adressing the online channel management issue from the standpoint of manufacturers has been published.

This study thus aims to fill this gap by comprehensively reviewing the existing body of knowledge, uncovering research gaps and understudied topics and leveraging them to propose a future research agenda. Therefore, after the application of strict inclusion and exclusion criteria the authors identify 92 valuable contributions published by 31 December 2021, which they consider as a dataset that will provide useful bibliometric insights to facilitate the evaluation of the extant body of knowledge. The authors, by adopting the bibliographic coupling methodology, observe that the body of published literature on the topic has fallen into three distinct yet interconnected blocks focusing on strategic marketing issues on manufacturervsretailer conflict, pricing policies and trade-offs among pricevsservices, and operational interactions and strategies between supply chain members. After exposing the bibliometric insights and identifying the three aforementioned categories, the study analysed a selection thereof describing twelve article subgroups addressing specific subtopics. Finally, the analysis yielded research propositions from the shortfalls identified in each subgroup plus a final, more comprehensive research proposition proposing further theoretical advancement.

## Theoretical background

Since the initial years of the twentieth century, online commerce has been gaining ground. Consequently, scholars have embarked on associated research to comprehend its potential and its corresponding business opportunities. Mahadevan (2000) distinguishes the three types of business models comprising the digital economy at the turn of the century: portals, market makers, and product/service providers. The primary focus of a portal is to create a network of people interested in learning more about a certain product or service. An example of an internet portal, as intended by Mahadevan (2000), could be Yahoo.com. Like a portal, a market maker, following Mahadevan’s (2000) perspective, connects buyers with sellers of goods and services, with the difference that the market maker requires more specific knowledge. An additional example is Ebay.com. Finally, product/service providers interact directly with their clients when conducting an online commercial transaction.

Mahadevan (2000) used Amazon.com as an example of the category of service providers. Indeed, in the early 2000s Amazon was simply an online bookseller. However, Amazon itself remains an emblematic example of the transformation of its digital business model. In fact, in 2006, the company launched the Fulfilled by Amazon (FBA) service (Zhu and Liu 2018). FBA revolutionized its business model from being a mere product provider to a hybrid product provider and market maker. In doing so, the company joined eBay as a market maker of choice connecting sellers and consumers online. From there, the interest of researchers in capturing this peculiarity grows. From that moment onwards, scholars inevitably began to pay attention to these types of platforms, called two-sided or multi-sided platforms (Hagiu 2007; Hagiu and Wright 2015; Tan et al. 2015). Indeed, several studies have embraced the capabilities perspective to assess the success of digital platform business models, referring to companies such as Ebay or Alibaba (Fuchs et al. 2011; Tan et al. 2015). In particular, Tan et al. (2015) analysing information systems capabilities through the Alibaba case study, observes that those required to maintain a successful platform are varied according to the maturity of the multisided platform itself, and these also evolve along with the platform. Subsequently, Teece (2017) used a four-stage model—Birth, Expansion, Leadership, and Self-Renewal—to examine digital platform lifecycle needs in terms of detecting, seizing, and converting high-level dynamic capability categories. The newborn phase emphasizes generative sensing and planning-stage seizing, but as the platform expands and stabilizes, it emphasizes "seizing" activities and modest modifications. Platform renewal involves recognising future possibilities, producing new platform and business model concepts, developing them alongside the present business, and finally undergoing a substantial transition to restart the platform lifecycle.

On the other hand, given the rising popularity of the e-commerce channel, researchers from different fields have begun to explore what capabilities traditional businesses need to implement a successful e-commerce business. As is common, the capability investigation frequently relates to the RBV, which asserts that resourceful companies absorb capabilities that help them achieve a competitive advantage, resulting in effective long-term performance (Barney 1991).

Following the RBV perspective, Gregory et al. (2019) argued that the investment of significant monetary but also headcount resources can subsequently sharpen specific marketing and communication capabilities in improving distribution effectiveness in the online channel. In support of this, the empirical study by Elia et al. (2021) identified the e-commerce manager as a true corporate resource, whose expertise and capabilities improve business performance such as internalization. On the other hand, three types of individual capabilities can be identified in the literature; namely, managerial capability (Khanin et al. 2022), talent capability (Santana and Díaz-Fernández 2022), and technical capability. These individual factors are necessary to achieve a successful e-commerce performance. Pivoting the focus from the individual level to the corporate level, other studies identify organizational capabilities, such as IT capabilities, big data analytics capabilities and strategic flexibility, data oriented organizational culture; these are required by companies regardless of their business domain to have performative e-commerce (Akter and Fosso Wamba 2016; Karaboga et al. 2022; Saini and Johnson 2005). Additionally, Cui and Pan’s (2015) qualitative investigation based on the RBV argued that, depending on the e-commerce implementation’s stage of maturity, the dedicated resources must increase or decrease, but especially must be orchestrated in the most appropriate way. They subsequently identify the focal capabilities that a manufacturer could face specific to every e-commerce maturity phase. In the early stages, sensing and responding capabilities are dominant, followed by the capability of cooperation and, in the most advanced phase, the capability of innovation. Indeed, scholars’ association of digital capabilities and innovation management capabilities with antecedents of business success is not accidental (Rubio-Andrés et al. 2022).

Lastly, a lively and growing strand of research dealing explicitly with online channel management, especially from manufacturers’ point of view has developed. Pu et al. (2020) are among the few who attempt to provide a recent overview of this literature branch while introducing their work distinctly identifying direct selling, agency selling, and indirect selling (reselling) as possible online selling formats for maufacturers. Indeed, Pu et al. (2020) argue that although multiple studies are published, these use a biased view and are based on models that may emit some factors or analyse scenarios that are in any case not comparable or assumption based, despite considering similar factors. Faced with such a fragmented literature niche, the need emerges to map the state of the art in a systematic manner.

## Methodology

To offer the best possible understanding of broad topics of literature, considering the growing scientific production in the business and management domain, and in particular in e-commerce, we adopt a methodology that combines bibliometric insights followed by a systematic literature review, referring to innovative protocols recently employed in the field (Behera et al. 2019; Bresciani et al. 2021; Ciampi et al. 2022; Kraus et al. 2020). Bibliometric methods facilitate the effective synthesis and interpretation of vast volumes of bibliographic data, as well as the detection of research streams in specific fields (van Eck and Waltman 2010), whilst simultaneously avoiding potential bias typically generated by the subjective interpretations characterizing the exclusive recourse to systematic literature reviews (Zupic and Čater 2015). Combining bibliometric methods flanking the review of the literature provides an opportunity to offer a shrewd and thorough systematic outcome. Specifically, we adopted the bibliographic coupling methodology relying on VosViewer 1.6.10 algorithm for which two contributions are considered to be coupled if they have a third or more common studies in their bibliography (Van Eck et al. 2006; van Eck and Waltman 2014, 2010). This algorithm has proven to be useful and reliable to support the mapping of research fields and the recognition of research streams or trends (Boyack and Klavans 2010; Ciampi et al. 2022). This makes it a bibliometric methodology that is particularly suited to this type of research subject, which is increasingly studied but in a very fragmented manner (Pu et al. 2020), thus providing the opportunity to offer a shrewd and thorough systematic review.

### Data collection

A reproducible and rigorous process was followed to select the sample of papers to be the object of our analysis (Akter et al. 2019; Kraus et al. 2022; Marzi et al. 2020; Pellegrini et al. 2020).

In line with the literature (Ciampi et al. 2022; Tranfield et al. 2003), we performed our bibliometric analysis and literature review in six phases. First, based on the main definition of online selling (e.g., online channel, e-commerce, etc.) and manufacturers (e.g., vendor, producer) the following search query was developed:

TITLE-ABS-KEY ( ( manufacture*) OR ( vendor*) OR ( producer*)) AND ( ( e-commerce) OR ( electronic AND commerce) OR ( eCommerce) OR ( online AND sales) OR ( online AND channel)) AND ( strategy) OR ( manage*)).

The Scopus database, which is considered an ideal scientific database for systematic literature reviews (Falagas et al. 2008; Kraus et al. 2020), was used to perform our search. In particular, beyond benefiting from a great reputation, Scopus provides greater coverage than the competitive Web of Science database, covering almost all (99.11%) of the journals included by its competitor (Singh et al. 2021). The “*” operators were used as jolly characters to include as many lexical variants as possible (Ciampi et al. 2022).

The implementation of our search query (the second phase of our analysis) allowed us to select an initial dataset of 2,565 papers. In line with the literature (Delgado García et al. 2015; Gupta et al. 2019; Kraus et al. 2020; Voester et al. 2017) and considering the managerial perspective of our review, we selected only articles or reviews from peer reviewed journals in English that had already been published or were in press by 31 December 2021 and belonging to business, management, and accounting scientific areas, refining the sample at 496 papers. Furthermore, in order to ensure the exclusive analysis of articles with satisfactory scientific value (Kraus et al. 2022), the authors decided to exclude all articles published in journals not included in the Academic Journal Guide 2021 released by the Chartered Association of Business Schools (ABS) reducing the papers list to 367. The search was also implemented in the Web of Science database for cross-validation purposes, without finding any other relevant articles.

**Fig. 1:**
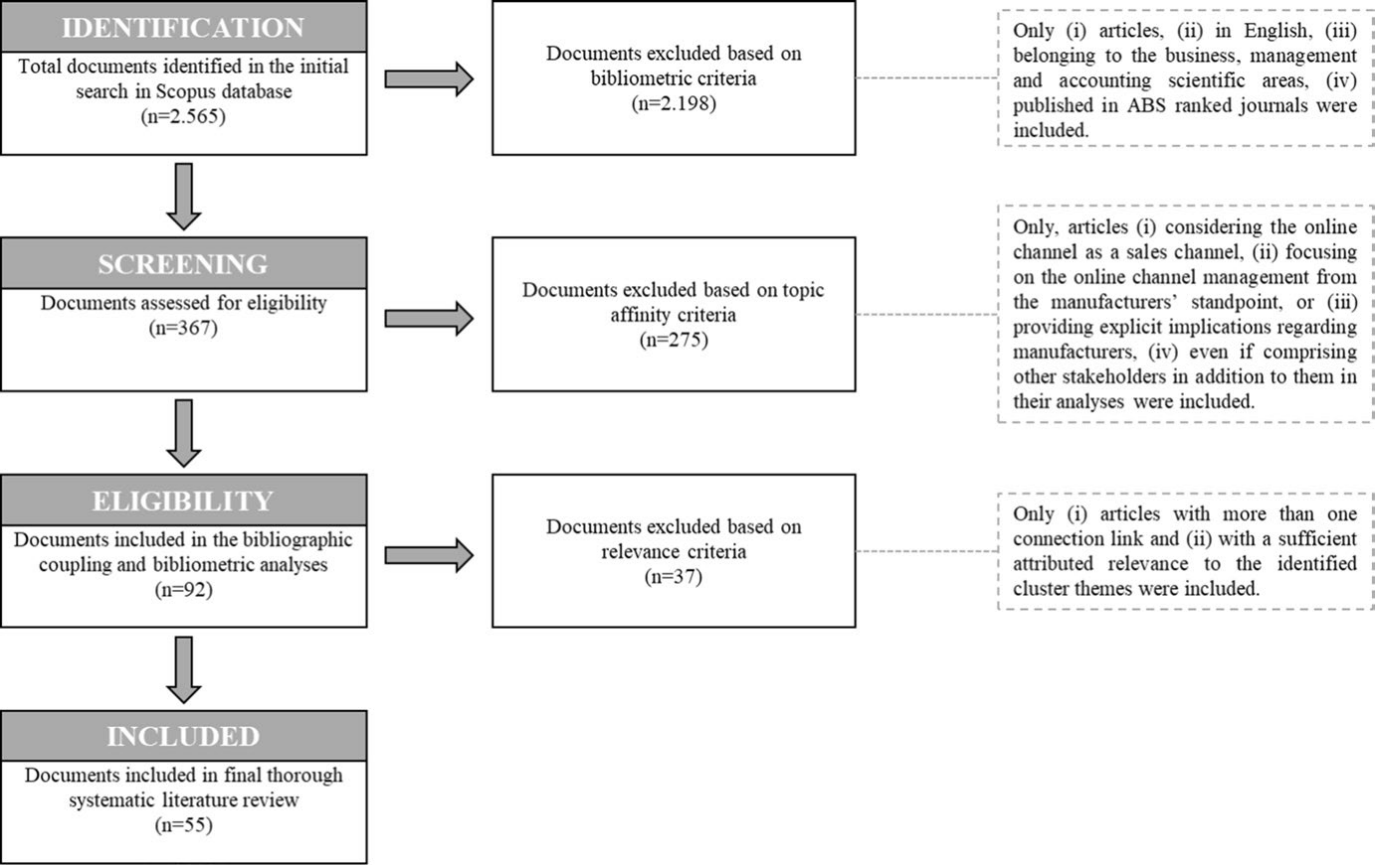
Selection protocol summary

In the following phase of our analysis, three out of four authors performed an autonomous reading of each of the 367 documents to independently analyse their relevance (Akter et al. 2019; Akter and Fosso Wamba 2016; Tranfield et al. 2003). The strict inclusion criteria that the authors decided upon prior to independent reading are: (1) the articles must focus on the managerial standpoint of e-commerce management by the manufacturers; (2) the articles must intend to provide implications for manufacturers; (3) including manufacturing companies in the analysis sample is not sufficient if the outcome does not propose peculiarities about them; (4) articles comparing manufacturers and other stakeholders can be included as long as these propose explicit implications about manufacturers; and (5) e-commerce is considered by the article as a sales and advertising channel, or only as a sales channel, but not only as an advertising channel. To measure the level of alignment between the results obtained through these three individual selection processes, Krippendorf’s alpha coefficient was calculated. It was greater than 0.80, thus supporting the robustness of our selection protocol. Many papers were excluded because they included the word “manufacturer” in their abstract, but they mainly focused their attention on retailers instead of manufacturers. Others were simply consumer oriented and not firm focused. Some papers were excluded because they did not delve into online channels even if the abstract included the word “online” with the word “sales”. This selection process reduced our dataset to 92 papers (see Fig. 1), which deeply analyse the online channel management topic from manufacturers’ perspectives.

In the fourth phase, to analyse the structure and evolution of the literature object of our review, some relevant bibliometric indicators were calculated and interpreted (Todeschini and Baccini 2016). Subsequently, thanks to its bibliographic coupling algorithm, VosViewer allowed us to build a graphical map in which each sphere represents a paper, and the papers are split into clusters as a function of the similarity of their references (van Eck and Waltman 2014, 2010). The resulting cluster structure represents a powerful instrument to interpret the literature contained in a scientific dataset and the research streams characterizing that literature (van Eck and Waltman 2014, 2010). As a result of our similarity analysis, 84 papers were found to be connected in terms of at least more than one shared reference, forming a graphical structure composed of three clusters whose configurations appear to be quite well defined (see Sect. 4).

In the fifth phase, always following the best methodological practices (Ciampi et al. 2022) and with the aim of focusing on the most relevant papers and making the review as significant as possible, the authors autonomously attributed a relevance score to each of these 84 papers as a function of their significance for the main topics touched upon by each thematic cluster. This final step allowed us to select a restricted dataset composed of 55 papers (65% of the total dataset). Figure 1 summarizes the selection protocol that led to the final sample of articles considered.

In the final step, a systematic review of these 55 papers was performed (Gaur and Kumar 2018; Tranfield et al. 2003) to explore and connect the themes analysed, investigate the most relevant connections among the studies and the thematic clusters, discover the most relevant understudied topics, and propose a future research agenda.

## Bibliometric insights

**Fig. 2:**
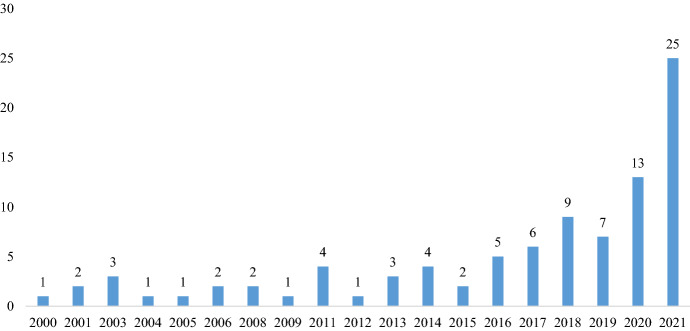
Papers per year

In Fig. 2 and Table 1, we present the dynamics of some bibliometric indicators that we consider useful for analysing and interpreting the structure and evolution of the object of our review. Starting from 2016, the number of publications per year has grown exponentially, demonstrating the recent increase in the topic’s scientific interest, in parallel with the increasing growth in e-commerce sales worldwide, which rose from 1.8 trillion $ in 2016 to 5.2 trillion $ in 2021 (Chevalier 2021).

**Table 1:** Papers per country, author, and journal

*Studies per country (top 10 countries)*			
China	49	Netherlands	3
United States	33	South Korea	3
Canada	7	Switzerland	3
Hong Kong	7	Taiwan	3
India	3	United Kingdom	3
*Studies per author (top 10 authors)*			
Yan R	10	Zhang X	3
Pei Z	9	Chen YJ	2
Chen J	3	Cheng TCE	2
Dan B	3	Chun SH	2
Pu X	3	Ghose S	2
*Studies per journal (top 10 journals)*			
International journal of production economics	10	Production and operations management	6
Journal Of retailing and consumer services	9	Industrial management and data systems	4
International transactions in operational research	8	Transportation research Part E: logistics and transportation review	4
Electronic commerce research and applications	6	Industrial marketing management	3
International journal of production research	6	International journal of electronic commerce	3

Table 1 shows that the number of authors with a significant volume of publications is relatively high, as are the number and variety of journals that published the greatest number of studies.

The main specific scientific fields covered by the most relevant journals are production, operation management, and information systems. Among the most prolific journals, the presence of operations and production is predominant (international journal of production economics, international transactions in operational research, international journal of production research, production and operations management), followed by specific e-commerce (electronic commerce research and applications, international journal of electronic commerce) and by marketing journals (journal of retailing and customer service, industrial marketing management). The distribution of studies per author reveals several authors (Yan R. and Pei Z.) who profoundly investigated the topic with more than eight contributions, followed by a quite large and varied group of twenty productive scientists with more than one single contribution (of which four had published more than two). Country wise, the distribution reveals that 53% of the scientific production comes from China and 20% from the United States, which is understandable since these two countries respectively represent 52% and 19% of the total global e-commerce market (von Abrams 2021).

**Fig. 3:**
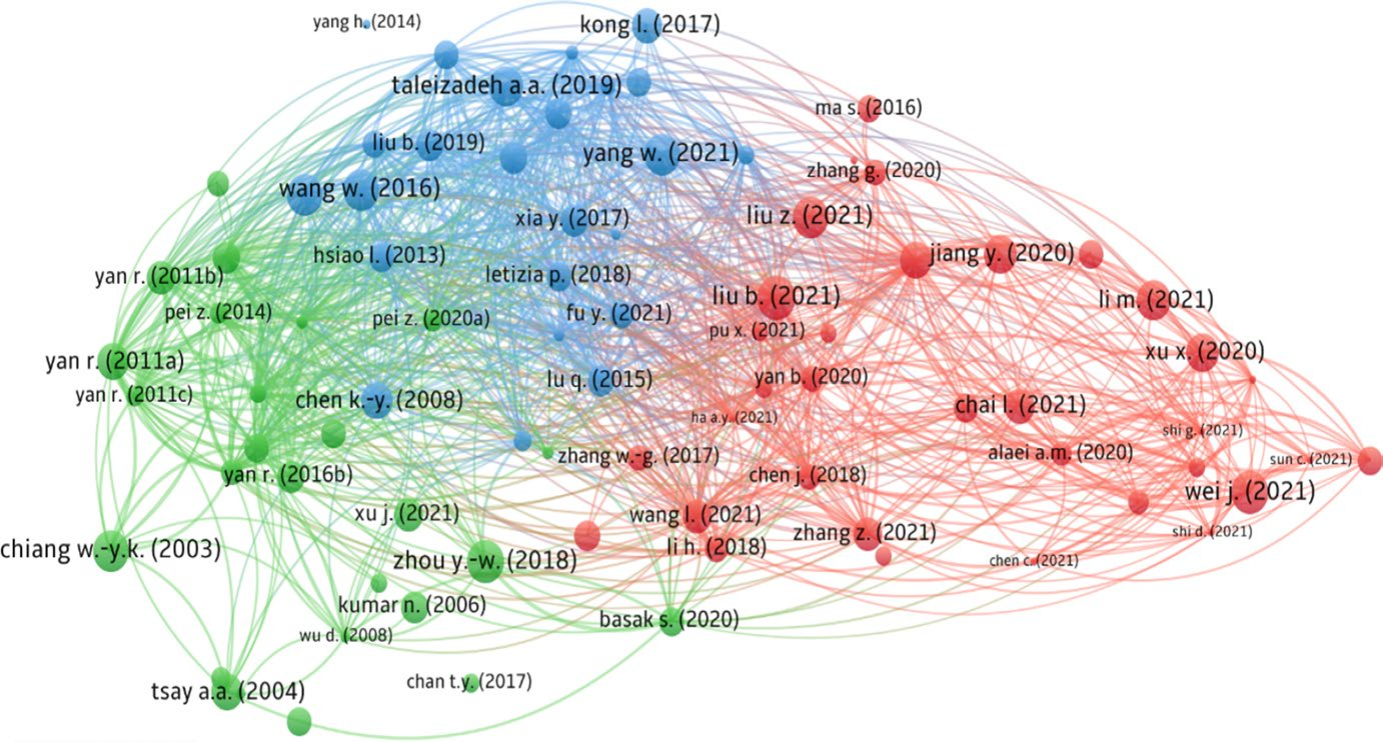
The clustering structure emerging from the VOS analysis

The clustering structure resulting from the similarity analysis performed by using VosViewer 1.6.10 software (Fig. 3) reveals the presence of three thematic clusters whose configurations appear to be outlined.

The papers belonging to the green cluster (the one that, on average, includes the oldest set of papers) concern the strategic marketing issues within the manufacturervsretailer conflict. The blue cluster aggregates work that pay attention to pricing policies and the pricevsservices trade-off. Finally, the red cluster assembles studies (the most recent, on average) focused on operational interactions and strategies between supply chain members.

## Systematic literature review

In the following sections, we present the results of our literature review based on the bibliographic coupling bibliometric analysis. Table 2 summarizes the main thematic areas that were the object of analysis within each cluster.

**Table 2:** Main topics per cluster

Green cluster: strategic marketing issues under manufacturer vs retailer conflict		Blue cluster: pricing policies and price vs services trade-off		Red cluster: operational interactions and strategies between supply chain members	
Main topics	References	Main topics	References	Main topics	References
Market structure and related strategies	Karray and Sigué, (2018); Kumar and Ruan (2006); Xu et al. (2021); Yan et al. (2011)	Pricing and online channel strategic choices	Chun and Park (2019); Taleizadeh et al. (2019); Wang et al. (2018); Wang et al. (2016)	The influence of different costs in online distribution and manufacturer choices	Chen et al. (2018); Li et al. (2018); Liu et al. (2021b); Sun and Ji (2021); Wang et al. (2021)
Information and related asymmetries	Lynch and Ariely (2000); Wu et al. (2008); Yan et al. (2016a); Yan and Pei (2011)	The role of consumer services	Chen et al. (2008); Liu et al. (2019); Luo et al. (2016); Xu et al. (2012)	The consequences of the spillover effect on supply chain members	Chai et al. (2021); Chen et al. (2021); Dan et al. (2021); Shi et al. (2021a)
Marketing and Advertising in the online channel	Chiang et al. (2003); Pei et al. (2014, 2020); Pei and Yan (2013)	Product return and channel management	Kong et al. (2017); Letizia et al. (2018); Xia et al. (2017)	O2O strategies and the benefits of integrating physical channel	Jena and Meena (2021); Jiang et al. (2020); Li et al. (2021); Liu et al. (2020); Zhang et al. (2021b)
Direct coordination and monetary incentives	Tsay and Agrawal (2004); Yan et al. (2011); Yan et al. (2016b); Yan et al. (2019)			Different online sales formats and platform approaches	Liu et al. (2021a); Pu et al. (2021); Shi et al. (2021b); Wei et al. (2021); Xu et al. (2022)
				How logistics levels can affect manufacturers and consumers	Alaei et al. (2020); Wang et al. (2019); Xu et al. (2020); Yan et al. (2020); Zhang et al. (2021c)

### Green cluster: strategic marketing issues within the manufacturer vs retailer conflict

#### Market structures and related strategies

A fair amount of scholars have explored how market structure pertains to the relationship between manufacturers and retailers. In studying this phenomenon, primarily using game theory methodology, they build models that include a manufacturer and a retailer in a market. For the market structure, many authors refer to the consumers comprising it. For example, Kumar and Ruan (2006) divide the market into brand-loyal and store-loyal consumers. Based on this discrimination, the manufacturer is better off entering the online market directly when most consumers are brand loyal. Karray and Sigué (2018) instead propose another classification of consumers, considering whether they are more (online buyers) or less (offline buyers) inclined to buy online based on the trade-off between prices and the service offered. Their model predicts that as the number of online buyers increases, the manufacturer should increasingly opt for a transactional website rather than an informative one and vice versa. On the other hand, the retailer appears to continue to earn a lesser income despite the introduction of an e-commerce website. Another perspective is provided by Yan et al. (2011), who refer to the degree of cooperation between the manufacturer and the retailer as a market structure. Their model confirms that in the case of non-coordination (neither allocation of marketing resources nor logistics nor prices), the manufacturer always yields significant profits. However, in a scenario of cooperation among both players, the market value would rise, generating a win–win situation. Finally, Xu et al. (2021) attribute an active role to the manufacturer’s influence on the market structure; their elaboration argues that when a manufacturer starts to sell directly online, it can afford to lower prices, thus increasing consumers’ purchasing power, thereby creating a consumer surplus and an increase in social welfare.

#### Information and related asymmetries

A great advantage of online shopping under consumers’ perspective is the possibility of lowering the cost of acquiring information. Consumers can find transparent information in just a few clicks without having to visit a retailer and relying on a salesperson. In addition, consumers are given the opportunity to compare offers and prices from different shops quickly and easily. Indeed, Lynch and Ariely (2000), analysing the sales of two wine retailers, observed that this has beneficial effects by stimulating price competition when there is an overlap of offers between shops. Moreover, the higher the possibility of comparing the same wine among retailers, the higher manufacturers’ chance of increasing their market share. On the other hand, it is true that not all products have the same level of availability of information online. Indeed, this may influence a manufacturer’s choice to sell to a specialized e-tailer, as well as to a traditional retailer. Wu et al. (2008) suggest that a manufacturer would only optimally approach different resellers on the two channels when the value of product information is much higher digitally than physically. In this manner, the e-tailer can afford to maintain a price premium over the physical without causing cross-channel price competition and price erosion. Information level also plays a crucial role in the direct relationship between a manufacturer selling online and their independent retailer. In a multi-channel competition, both players will try to maximize their pricing policy. However, in order to best maximize the pricing policy, it is necessary to ascertain the extent of the demand, the cost of the product, and its fit with the online channel (Yan and Pei 2011). If there is no information sharing, the retailer will always know more than the manufacturer who does not know the retailer’s turnover (and their online/offline split) but only its own online turnover, while the retailer will always know at least its supplier’s selling price. Therefore, the manufacturer needs to find a key to stimulate information sharing by the retailer. Yan et al. (2016a) suggest that incentivising retailers by providing them with advertising contributions and proposing shared marketing operations is a good driver to increase information sharing. However, the other side of the coin is the threat of data manipulation by the latter.

#### Marketing and advertising on the online channel

Since the early 2000s when big brand manufacturers such as Nike, Dell, HP, Estee Lauder, etc., began to sell directly online, the question arose as to whether and how sensible it was to allocate marketing budgets to direct sales, although at first the numbers of direct online sales were especially derisory in relation to total turnover. If one were to focus only on the direct ROI of related advertising campaigns, one would not see anything positive. However, Chiang et al. (2003) argue that the more a brand advertises their direct online channel, the more the perceived risk on the part of the retailer increases. When accompanied by a decrease in the manufacturer’s wholesale price, this dynamic results in a price reduction by the retailer with a subsequent increase in volume that can even compensate for a zero in online sales. However, it is not only online advertising that increases the value of supply chain profits. Rather, investing on a broader, more mainstream level and contributing to brand image boosts sales in both channels by reducing conflict and price erosion (Pei and Yan 2013). An effective and more targeted practice is supportive advertising. To mitigate channel conflict, especially when the retailer has significant bargaining power, the manufacturer may want to create campaigns about their own brand but tailor them to be activated through their retailer. This increases total product sales, offsets the customer switch (from the retailer to the brand’s new direct online channel), increases the retailer’s sales, and boosts its "sentiment" (Pei et al. 2020, 2014). Nevertheless, the fact that there is competition between several manufacturers selling online should also not be underestimated. Chan et al. (2017) confirm the conclusions of previous studies about lowering prices and mitigating conflict with retailers. However, they also argue that the greater the increase in advertising, the greater the price sensitivity developed in consumers, which consequently leads to a greater price battle on the online channel between manufacturers. The subsequent trade-off of margin erosion vs. more sell-out is not necessarily positive for all brands on the market.

#### Direct coordination and monetary incentives

In addition to advertising, scholars have analysed strategic methods to maximize profits by mitigating conflict between channels. Manufacturers can pay a commission to the retailer for each customer that is redirected to its direct online channel. Conversely, the scheme may involve the brand’s own website redirecting sales to the retailer who then plays a fulfilment role. This type of collaboration ensures that the two players can each focus on a task related to their competitive advantage (Tsay and Agrawal 2004). Consumers will be "attracted" through the more cost effective method and/or are served in the more cost effective method. Some research suggests that a direct monetary incentive may positively affect profits and reduce channel conflict. Even when differentiating between the offerings that are sold online or distributed via the manufacturer to the retailer, the latter will still be denied some of its market share because of the alternative goods sold online. To avoid tensions, especially if the retailer is influential, scholars envisage a profit-sharing scheme, in which manufacturers can agree in advance to share some of their newly generated online revenue with the retailer (Yan et al. 2016b, 2011). However, these solutions bring with them several barriers in the real world, and there is often reluctance on both sides to adopt them. Nevertheless, Yan et al. (2019) argue that the implementation of a newer method, namely reward points, can be either an effective solution in its own right or a complementary one to encourage profit sharing.

### Blue cluster: pricing policies and price vs services trade-off

#### Pricing and online channel strategic choices

Scholars argue that, whether in a single retailer-manufacturer channel or a multi-retailervsmanufactures channel, it is inefficient for the manufacturer to focus on differentiation in product distribution between channels to secure profits (Taleizadeh et al. 2019; Wang et al. 2016). The operating costs associated with this choice are often overwhelming. Conversely, converging product availability in each channel increases the retailers’ disadvantage in rivalling the manufacturer due to the wholesale markup they have to pay for the products. To drive its profit maximization, the manufacturer may thus adopt a price-matching strategy, aligning its direct channel with the price of the traditional retailer, subsequently pushing the latter to lower its prices. The cheaper the product, the more competitive it will be (Chun and Park 2019). What is more, the manufacturer also has a bargaining chip in its hands, allowing itself to raise prices as it pleases, yielding a dominant position. Retaining an online direct sales channel seems necessary and convenient for manufacturers. R. Wang et al.’s (2018) study of three different scenarios, in which the online channel is done either by the brand alone, the retailer alone, or both, corroborates these findings. Although considering a zero cost of channel opening, the wholesale price discriminator is a determinant in guaranteeing higher profits for the manufacturer in each contingency. In specific contexts (as noted in the previous section), the manufacturer may decide to outsource the fulfilment of products sold on its online channel to its retailer, who receives a percentage of sales. Beyond the cost efficiency argument, scholars argue that if the market share of the online channel is high enough, besides mitigating channel conflict, it can lessen the retailer’s tendency to lower prices on the product and thus prevent excessive price erosion for the whole market (Xu and Qiu 2020). Finally, the recent study by Fu et al. (2021) analyses the variation of a manufacturer’s retail price according to the type of e-commerce platform it uses. The study evidences that the quality of customer service and especially the platform’s commission fee are key factors positively correlated with the increase (or decrease) of the retail price.

#### The role of consumer services

Consumer service is another critical factor impacting the channel-retailer relationship. It is intuitive to think that the retailer should focus on pre-sales services to compensate for the price gap with the manufacturer’s online channel. However, this brings with it the risk of free-riding by consumers, leading them to use the retailer’s pre-sales services and then finalize their purchase online. The scenario presented by Luo et al.’s (2016) predictive model presents how an increase in customer service performance leads to an increase in sales and, in turn, to market growth. However, it also presents the risk that in the tentative effort to combat the online channel’s low prices, the retailer will cut back on its customer service, thereby shrinking the market. Accordingly, the manufacturer should cooperate with the retailer to increase customer service and protect the market value (Luo et al. 2016). Chen et al. (2008) stress how different services for consumers can contradict themselves, such as the online channel’s service of providing a vast catalogue of products with the disadvantage of having a lead time due to shipping delays. The manufacturer must therefore be able to discriminate the types of consumers based on their waiting time acceptance or based on its products and opt to distribute its goods as effectively as possible between online channels and retailers (Xu et al. 2012). A straightforward weapon for the manufacturer to increase consumers’ acceptance could be the pay-on-delivery service. Allowing consumers to pay on delivery greatly diminishes their resistance to online purchases (Liu et al. 2019).

#### Product return and channel management

An issue linked to customer service is the return rate. In many market verticals, the higher the customer service, the lower the product return rate (Letizia et al. 2018; Xia et al. 2017). In this respect, manufacturers must carefully consider this variable when deciding whether or not to enter the online channel. Nonetheless, in some product categories where recycling and remanufacturing are possible, the manufacturer must also consider service costs and recycling costs in their pricing policies. There is likely a positive correlation between the level of customer service and the ease of obtaining more returns (of old/exhausted products) for recycling. In some cases, the manufacturer may decide to lower its wholesale price to the retailer, allowing it to have a sufficient margin to introduce an efficient product return service policy (Kong et al. 2017). The savings coming from the avoidance of direct remanufacturing costs reward the manufacturer’s loss from B2B sales.

### Red cluster: operational interactions and strategies between supply chain members

#### The influence of different costs in online distribution and manufacturer choices

Some scholars consider how costs related to the introduction of online distribution can influence choices and the relationship between manufacturers and retailers. Chen et al. (2018) found that, even if the setup cost of introducing an online channel is high, the manufacturer will establish an online channel to dissuade the retailer from introducing a discount store. Other types of costs are examined by Wang et al. (2021), who argued that the manufacturer is motivated to introduce an online channel as a ‘margin-oriented strategy’ when the consumer’s hassle cost of online shopping (finding the product online, ordering it, and waiting for delivery) is relatively low, and transportation cost (of travelling to and from the retailer) is relatively high. In addition, Liu et al. (2021b) demonstrated how the unit manufacturing cost of a new product also affects whether or not a manufacturer enters the online channel. They argued that if the unit cost of manufacturing the new product is below a certain threshold, the manufacturer will be unwilling to engage in remanufacturing, consisting of returning a used product to at least its original performance with a warranty that is equivalent to or better than that of the newly manufactured product.

Moreover, when there are sufficient returns, the retailer’s channel selling cost does not affect the manufacturer’s remanufacturing decision. Finally, Sun and Ji (2021) revealed how technology costs can impact manufacturer and channel performance. Their elaboration, in which an online platform provides IoT infrastructure and a manufacturer sells its products on the platform, surprisingly found that both manufacturer and channel performance could be negatively affected by an increase in the value of IoT technology in certain situations. If the manufacturer invests in this new IoT technology, contrary to what might be expected from common intuition, the manufacturer will be worse off.

#### The consequences of the spillover effect on supply chain members

The spillover effect indicates how the spread of buying behaviors between online and offline channels significantly influences manufacturers’ expectations and decisions in choosing channels. Chen et al. (2021) explored how a manufacturer’s bargaining power and the online spillover effect can positively or negatively influence the introduction of an online channel. They inferred that reselling will be unprofitable when the online spillover effect is significant, and the manufacturer has weak bargaining power. On the contrary, when the online spillover effect is weak, the online channel will profit the manufacturer. Conversely, when the online spillover effect is strong, sales on the online channel significantly affect the offline channel sales. Another perspective is provided by Shi et al. (2021a), who investigated the problem of supply chain members facing strategic decisions regarding whether to introduce online marketplace platforms in addition to their existing brick-and-mortar sales channels. In this context, where the manufacturer sells only through the retailer, and the retailer sells through both a physical and a digital marketplace, the cross-channel spillover effect can improve the equilibrium for both supply chain members. Other scholars have demonstrated that the spillover effect is a significant feature that must be considered when manufacturers and retailers collaborate in an O2O or showrooming context (Chai et al. 2021; Dan et al. 2021). Chai et al. (2021) considered that the service spillover effect (a factor that affects how O2O retailers and manufacturers operate) will increase both total demand and wholesale price for a manufacturer, which will improve its profitability. Moreover, they showed how spillover intensity can increase retail prices (online and offline) and significantly affect retailer profit and service strategy. By contrast, Dan et al. (2021) analyzed the online manufacturer’s optimal collaboration strategy for establishing a physical showroom by comparing its profits in different cases. They argued that the competing retailer provides a lower service level than the noncompeting retailer, which leads to a negative competition effect and a positive spillover effect in the competing case; the trade-off between the two effects decides the online manufacturer’s optimal strategy. In addition, when the positive spillover effect dominates the negative competition effect, the online manufacturer prefers to partner with the competing retailer and strategically sets a high price.

#### O2O strategies and the benefits of integrating the physical channel

The emergence of new forms of Online-to-Offline (O2O) commerce, combined with the opportunity for interaction and integration with the phenomenon of showrooming, is becoming an increasingly common practice, meaning that a good number of scholars have begun to study its dynamics and strategies (Jiang et al. 2020; Jena and Meena 2021). Examining retailers’ BOPS strategies (Buy-online-and-pick-up-in-store) and analyzing the service value of the physical store, Jiang et al. (2020) consider two key factors, namely the manufacturer’s strategies on pricing and service value. Their model argues that, in a centralized dual-channel structure, the retail service value increases along with the offline selling price, while, in a centralized omnichannel structure, both online and offline prices increase with service value. However, in a decentralized omni-channel and dual-channel supply chain, the traditional channel retail price is significantly and positively correlated with service value. Another perspective is provided by Jena and Meena (2021), who investigate the impact of test-in-store-and-buy-online (TSBO) and competition retailing on overall supply chain profit. They observe that retailing benefits all supply chain players under the integrated channel. In addition, many researchers have focused on manufacturer-retailer cooperation to test the effects of the physical showroom (Li et al. 2021; Zhang et al. 2021b; Liu et al. 2020). Li et al. (2021) explored how the commission rate and convenience coefficient have different impacts on cooperative advertising and the profits of supply chain members in two different strategies. Their results demonstrate that, in one strategy, the commission rate substitutes the incentive effect of cooperative advertising and positively impacts the profits of supply chain members and vice versa. Zhang et al. (2021b), by contrast, suggest that the impacts of introducing a competing product (from a retailer with service decisions) on the profits of the online producer and the retailer are inconsistent. Their model explains that when product competition harms the retailer but benefits the online manufacturer, the latter would encourage the retailer to introduce the competing product and vice versa. Finally, Liu et al. (2020) demonstrate that, regardless of channel structure, a display showroom can generate benefits for the manufacturer, the retailer, and the whole omnichannel supply chain. The underlying reason for this is that service cooperation helps ease price competition and reduces the double marginal effect between offline and online channels.

#### Different online sales formats and platform approaches

The varying approaches to channel choice strategy from the manufacturer’s perspective have attracted the attention of many scholars (Pu et al. 2020; Wei et al. 2021; Xu et al. 2021; Shi et al. 2021b; Liu et al. 2021a). Pu et al. (2021) considered the manufacturer’s marketing and pricing strategies to understand which channel is the best to adopt. Their findings support the notion that the manufacturer should select the online agency selling mode when the third-party platforms have a low commission rate. Otherwise, the manufacturer should opt for the online reselling method if the commission is high. Additionally, Wei et al. (2021) explored the optimal scenario between reselling or agency selling choices for a manufacturer dealing with two different e-tailers. The most profitable scenario is when the manufacturer engages the two with an agency selling format and charges equal fees. This scenario remains valid independently of the two e-tailers’ channel roles and the difference in the two e-tailers’ market shares. Shi et al. (2021b) elaborate on four other scenarios in which a manufacturer and its retailers can both consider the adoption of agency selling through a third-party marketplace platform. As long as channel competition is not too fierce, the manufacturer is better off incorporating a third-party marketplace to boost sales. If the retailer also adopts it, the benefits of increased sales strongly outweigh channel cannibalization. Other scholars have pointed out that other exogenous factors can influence sales and channel selection (Liu et al. 2021a; Xu et al. 2021). Indeed, Liu et al. (2021a) argued that competitive intensity and order-fulfilment costs are two driving factors that significantly influence the manufacturer’s strategic choice of selling contract mode. Specifically, for a given competition intensity, with rising order-fulfilment costs, the preferred method for the manufacturer changes from the online marketplace model to the hybrid mode (both direct selling and marketplace modes) and then to the reseller model. This intuition lies in the interaction of the transfer of the pricing rights and the responsibility for order fulfilment. Finally, considering consumers’ environmental awareness, Xu et al. (2021) found that increasing the cross-channel effect (CSE) can positively influence the level of carbon reduction, production quantity, and profits when manufacturers adopt an agency selling format. Marketplace mode provides the manufacturer with more earnings than reselling mode and induces the manufacturer to adopt greener technology if the CSE is high, contrarily vice versa. The consequent underlying reasons are that the carbon tax regulation stimulates greener product production, and manufacturers counter the cost increases accompanying these new greener productions by increasing retail prices and overall market value.

#### How logistics levels can affect manufacturers and consumers

Many scholars have explored the differing service levels that can benefit or disadvantage manufacturers’ decisions on the choice of online and offline sales channels, highlighting their effects (Alaei et al. 2020; Yan et al. 2020; Zhang et al. 2021c). Accordingly, Alaei et al. (2020) analyzed how return policies affect a manufacturer’s decision to choose between reseller and marketplace channels. Their elaboration argues that the manufacturer offering a return policy in their own channel (web-store) does not affect their choice. However, it impacts the amount of manufacturer profit in each channel. Furthermore, they assert that regardless of offering a return policy, as the coefficient of CSE increases, the manufacturers’ profits increase, whether they choose a reseller channel or marketplace channel. Yan et al. (2020) analyzed the optimal pricing and logistic service level under different potential models. They observed that the manufacturer’s online channel opening can indirectly stimulate consumers to buy through traditional retail channels. Indeed, the manufacturer’s online channel is more conducive to improving the profit of the whole supply chain than the retailer’s online channel under the dual-channel structure. Finally, multiple channels can provide consumers with the lowest retail price, one that is also most beneficial to the retailer. Zhang et al. (2021c) provided another standpoint in which the manufacturer, selling directly through its stores, suffers critical inventory misplacement problems in the direct sales channel if it chooses encroachment. Their investigation found that the retail channel’s misplacement problem encourages the manufacturer to encroach, compared to the direct channel. Moreover, if only the retail channel employs RFID (Radio-frequency identification; i.e., the most effective technology to eliminate inventory misplacement problems), the encroachment cost threshold increases with the RFID tag price; the opposite occurs if a direct channel is used. Finally, employing RFID technology also intensifies channel competition, thus the more intense the channel competition is, the more likely the manufacturer will be to encroach. Other scholars have explored several issues related to logistics and the level of supply chain coordination by considering cost-sharing and the power of platforms (Xu et al. 2020; Wang et al. 2019). Xu et al. (2020) explored how several factors play an essential role in the supply chain coordination problem, including the commission rate, platform power, and the consumers’ sensitivity to delivery time. Their insights indicate that the supply chain can be coordinated via wholesale price and cost-sharing contracts with considerable platform power. If not, it can be coordinated via a cost-sharing contract when delivery time sensitivity is relatively high. Further, if consumers’ sensitivity to delivery time is high and the platform’s commission rate is moderate, the optimal wholesale price and retail price change in opposite directions. Wang et al. (2019), referring to the development of small and medium-sized e-commerce platforms, discovered that capital constraints are an essential factor that restricts this development. In this regard, they explore the conditions for sharing service costs and loan strategy implementation from the manufacturer’s perspective and the e-commerce platform. They infer that when the manufacturer shares part of the service cost, the more self-owned platform funds involved, the more beneficial it is for the manufacturer, thus resulting in a highly advantageous method of relieving capital constraints.

## Discussion and research propositions

To inspire future research directions, and in line with the recent systematic literature review techniques (Ciampi et al. 2022; Pellegrini et al. 2020), we now present a series of clearly stated and straightforward research proposals based on the research gaps identified. Based on the literature review presented in section four, and seeking to guide the commission of future investigations regarding the online channel management from the perspective of manufacturers, twelve unique research proposals are outlined in the next sections, each addressing one or more of the research gaps identified in every subsection analysed so far. The proposals are not specific to particular industries or cases, but may have relevance in a variety of business or market contexts. Table 3 synthesizes the most relevant understudied topics that emerged from our review.

**Table 3:** The main research gaps per cluster

Green cluster: strategic marketing issues under manufacturervsretailer conflict	Blue cluster: pricing policies and price vs services trade-off	Red cluster: operational interactions and strategies between supply chain members
Addressing complex market structure dynamics	Pricing policies in complex multi-manufacturer scenarios	Investigating the reversal of the status quo: the retailer is empowered to be the leader in choice
Investigating the role of market intelligence tools	Exploring the bidirectional consumers’ free-riding phenomenon	Understanding spillover effects in complex scenarios
Deepening knowledge on the most common and effective brand-retailers advertising techniques	Exploring the bidirectional consumers’ free-riding phenomenon	Exploring reverse showrooming and newer technologically enabled strategies
Investigating on barriers to profit sharing adoption and new monetary incentives	Investigating market competitiveness affection over return rate policies	Going beyond profit dogma, exploring different online channel affordances
		Fulfilment partnerships with third-party online marketplace platforms

### Propositions addressing strategic marketing issues in a manufacturer vs retailer conflict

#### Addressing complex market structure dynamics

Studies investigating the effects of market structure over the manufactuer’s online channel strategies, either focused their attention on the consumers’ classification (Karray and Sigué, 2018; Kumar and Ruan 2006) or the market power split among manufacturer and retailer (Yan et al. 2011). Unfortunately, to date the literature has not yet explored the dynamics of coordination between channels based on realistic models (or primary data collection) where in a given market both retailers and manufacturers are multiple.

##### Proposition 1

*Researchers should expand current understanding of the market structure dynamics concerning a multiple manufacturer and multiple retailer context, basing their investigation on more realistic models or on primary data.*

#### Investigating the role of market intelligence tools

The issue of information availability and information asymmetry, both on the consumer side and on the manufacturer-retailer side, has certainly been of interest to researchers. Several studies have described how manufacturers compensate for the lack of information sharing by retailers and how to encourage them to share information (Yan and Pei 2011). To date, most manufacturers use the services of third-party companies to monitor market trends and sales, e.g., Gesellschaft für Konsumforschung (GFK) or Nielsen (Duch-Brown et al. 2017), while consumers use different types of price comparison tools and online information. At present, no study focusing on information sharing has taken these tools into account.

##### Proposition 2

*Future research could seek to better understand if, how, and at what cost the market intelligence tools provided by external providers influence the manufacturer-retailer relationship in managing the online channel.*

#### Expanding knowledge on the most common and effective brand-retailer advertising techniques

Scholars agree that cooperation in advertising between brand and retailer is an effective resource for manufacturers to increase the total demand for their products and mitigate channel conflict (Chan et al. 2017). Studies on this subject present the game theory methodology to establish models that explain the effect on the market as cooperative advertising practices increase. However, scholars have not focused on comprehensively identifying which cooperative advertising practices are currently being employed, and which are more effective than others.

##### Proposition 3

*Future studies focusing on the effects of manufacturer-retailer cooperation in advertising as a driver for maximising online channel profits could focus on the effectiveness of different existing practices.*

#### Investigating current barriers to profit sharing adoption and new monetary incentives

Several researchers have investigated the possibility of a situation in which a manufacturer adopts profit sharing to compensate for its retailer’s losses (Yan et al. 2016b, 2011). However, the same researchers agree that although this could be an optimal solution to channel conflict, it is a highly unlikely scenario in real life (Yan et al. 2019). Recently, research has therefore focused on other types of monetary incentives such as reward points or cashback for consumers but, to date, our knowledge about monetary incentives remains limited (Ballestar et al. 2019).

##### Proposition 4

*Researchers could invest in understanding how manufacturers can find ways to overcome the barriers that prevent them from profit-sharing with retailers, and whether alternative monetary incentives can mitigate these barriers or supplant this attempt outright.*

### Propositions addressing pricing policies and price vs service trade-off gaps

#### Pricing policies in complex multi-manufacturer scenarios

Scholars have analysed different scenarios in order to recommend the optimal pricing strategies that the manufacturer should adopt in the online channel. However, the different factors taken into account by the research to date, despite being manifold, have been evaluated on theoretical models in which only one manufacturer and one retailer are considered, or there are several retailers but still only one manufacturer (Taleizadeh et al. 2019; Wang et al. 2018, 2016).

##### Proposition 5

*Future research into manufacturers’ pricing policies in the online channel could consider complex scenarios where multiple retailers but especially multiple competitors are taken into account.*

#### Exploring the bidirectional consumers’ free-riding phenomenon

Mainstream research conducted on the significance of customer service has reached a consensus that it is in the manufacturer’s interest to incentivize the retailer’s customer service (Luo et al. 2016). The assumption is that online brands cannot offer the customer service of a physical store, but can take advantage of the free-riding of physical customers who browse in-store and buy online to take advantage of low prices. However, as looking for product information online has become more convenient and less costly for some customers, they might use the online channel for research and learning, but they end up buying the products in nearby offline shops. Moreover, they might "touch and feel like" the products in physical shops but switch to online shops for purchase. Therefore, free riding behavior could be bidirectional when the online channel and the traditional shop channel provide complementary services.

##### Proposition 6

*In the future, researchers should address the phenomenon of consumers’ bidirectional free riding between the retail channel and the direct online channel to ascertain if and under what circumstances it can affect manufacturer-retailers’ cooperation strategies.*

#### Investigating market competitiveness affection over return rate policies

A relevant factor considered by researchers is obviously the return rate. In fact, the lack of the possibility of touch and feel inevitably leads to returns. For example, Zalando, the leading online fashion website, has a return rate of 40% of its sales (Difrancesco et al. 2018). Extant research has revealed that, logically, the return rate affects the prices that manufacturers can afford to charge online, since returns are a cost. However, all scenarios analysed by the researchers involve only one manufacturer. It is thus possible that in a multi-manufacturer competitive environment, the primary objective of the player may not necessarily be profitability but rather gaining market share, therefore accepting a high number of returns and instead finding a way to recycle the product.

##### Proposition 7

*Future research should consider competitive scenarios and cost acceptance due to the return rate and the eventual possibility of recycling products.*

### Propositions addressing gaps on operational interactions and strategies between supply chain members

#### Investigating the reversal of the status quo: the retailer is empowered to be the leader in choice

Channel choice is an important factor that can lead to interaction or competition between manufacturer and retailer and, at the same time, the cost of the channel strongly influences their strategic decisions and operations. For this reason, much attention must be paid to the convenience of entering one channel rather than another. Indeed, scholars have extensively investigated the influence that factors, such as production, pricing, or the introduction of particular products or technologies, can have on manufacturers’ strategic channel choices from different perspectives. In turn, most of these studies assume that the manufacturer is a Stackelberg leader and the first mover in making decisions. Consequently, it would be interesting to explore the problem in a retailer-led SC, since many big retailers such as Walmart lead the SC and squeeze the profit of their manufacturers (Giri et al. 2017).

##### Proposition 8

*Future research could focus on costs and related strategic decisions, particularly considering scenarios in which the retailer is the Stackelberg leader since in several cases they are the one with the most bargaining power.*

#### Understanding spillover effects in complex scenarios

Studies show that the spillover effect is an increasingly important scenario to analyse between online and offline channels. Its impact spills over into several factors such as manufacturers’ decision-making processes or choice of optimal collaboration strategy, causing a resulting increase or decrease in profits. However, actual studies have not delved into how the impact of the spillover effect varies in different market contexts; for example, in a multi-stage gaming scenario, an asymmetric information structure, or in competitive environments, such as a situation in which the online manufacturer works with multiple retailers simultaneously, competitors and non-competitors, or the introduction of third-party platforms that may provide differentiated services, such as personalized recommendations. Further, the various service and store cost structures, which are neglected in these studies, may provide new information about the retailer’s service decision and, therefore, the manufacturer’s strategy.

##### Proposition 9

*Further research is needed to explore the spillover effect in complex scenarios, including with multiple retailers or multiple manufacturers.*

#### Exploring reverse showrooming and newer technologically enabled strategies

In an O2O business model, many studies focus on the interaction of retailers’ and manufacturers’ respective strategies on pricing and service value in the field of supply chain management. Indeed, the literature addresses the BOPS and TSBO strategies, but there are other O2O commerce strategies besides BOPS, such as buy online and consume offline or evaluate offline and buy online; these should thus be considered in order to evaluate potential alternative strategies. Moreover, new technologies such as AI entail new kinds of business model adaptations (Åström et al. 2022), and manufacturers are therefore also called upon to consider them.

##### Proposition 10

*Future research should therefore consider newer supply chain structures such as ROPS and reverse showrooming, as well as how new technologies could favour new business model adaptations.*

#### Going beyond profit dogma, exploring different online channel affordances

Scholars addressed the various approaches and platforms that enable manufacturers to sell online. Many aspects have been analysed, from platform costs to logistics costs to environmental costs (Pu et al. 2021; Xu et al. 2022). These have all been explored in relation to manufacturer profit maximization. However, other areas of the literature already partly advocate the notion that e-commerce holds peculiar affordances such as internationalization or the knowledge of consumer big data, as well as an impact on profits (Akter and Fosso Wamba 2016; Hossain et al. 2021). Nonetheless, no research has investigated what online channel approach best suits different kinds of e-commerce affordances.

##### Proposition 11

*Researchers should investigate the types of sales formats or digital platforms that are most suitable for the different types of strategic affordances related to the online channel beyond the profit motive.*

#### Fulfilment partnerships with third-party online marketplace platforms

Currently, important factors such as return policy, consumers’ sensitivity to delivery time, cost-sharing, logistic service level, or the relief of capital constraints are increasingly influencing the supply chain, so there is a need to delve into the benefits thereof and assess whether the implementation of additional services within the supply chain will bring greater benefits to the consumer or the manufacturer. In fact, online third-party marketplaces are now equipping themselves to provide logistical (and other) support to manufacturers. Amazon or Alibaba, for example, offer to handle logistics management entirely for their vendors on agreed products (Zhang et al. 2021a; Zhu and Liu 2018).

##### Proposition 12

*A further avenue for research could be to assess the impact and presumed convenience of the fullfilment partnership options from the point of view of manufacturers and market impact.*

### Proposition beyond the thematic clusters

The extensive systematic analysis of the literature performed subsequently highlights and outlines the various literary strands that have so far dealt specifically with the online channel management strategy on the part of manufacturers. Although Teece (2017) highlighted that capabilities are not strategies, the strategies are still rather dependent on the resources and capabilities of the manufacturers themselves. To date, however, the extensive stochastic and assumption-based modelling of this strand of literature has left little room for the aforementioned RBV and capabilities theoretical framing and its subsequent theory testing.

#### Proposition 13

*The next path for the literature to follow would be to centre in on the theoretical framing of e-commerce dynamics from manufacturers’ perspective and then eventually attempt to test their viability. The Resource Based View appears to be a valid proposal, although it is not necessarily the only one to consider.*

## Conclusion

In this last section, we establish a discussion from the theoretical and managerial perspective, followed by the disclosure of the research limitations and how we tried to overcome them, as well as a future research lines section in which we summarize the process that led us to identify thirteen literature gaps and in which we tabulate the identified thirteen propositions for future research.

### Theoretical contributions

The theoretical contributions of this study are threefold. First and foremost, this study relies on an ironclad protocol and a software that allows for an objective bibliographic coupling analysis, whereby we discover and analyse three different, albeit interconnected, thematic clusters, focusing on strategic marketing issues on the manufacturer vs retailer conflict, pricing policies and trade-offs among pricevsservices, and operational interactions and strategies between supply chain members. Secondly, through careful reading and analysis, the authors have identified twelve interlinked intra- and inter-clusters but distinct themes. Each of these can be interpreted as a specific perspective and point of view towards the management of the online channel by manufacturers. Third, our research proposes thirteen original research propositions regarding new research avenues to be followed and new managerial solutions to be designed and implemented in the field of e-commerce and online channel management. In particular, through the theoretical background section and the final proposition (No. 13), this study exposes a discrepancy between the body of literature that has generally dealt with the subject of digitization and online commerce, and the literature that has specifically dealt with online channel management from the manufacturers’ perspective. In fact, on the theoretical framing front, this second niche-specific literature neglects established theories such as RBV or the capabilities perspective. On the other hand, this particular literature has often approached, sometimes even subtextually, the growing theoretical construct of ‘coopetition’ (Bouncken et al. 2015) and highlighted some of its functional implications. Our modest opinion leads us to propose that there may be considerable room available in this genre of literature for further theory-based studies that empirically test the efficacy of the proposed theories.

### Implications for practice

The study’s findings have implications for the practical implementation and design of managerial strategies for brands and manufacturers willing to enter, or having already encroached, the online sales channel. First, we emphasize how online channel policies depend on several factors that go beyond the classic marketing mix concept. In addition to focusing on the product, price, place, and promotion thereof, this paper highlights how manufacturers’ brands or product managers have to take into consideration the fundamental value of the relationship factor with retailers. In fact, the spillover effect, customer service, the choice of sales format, information asymmetry, and logistics issues underlined in this literature review all imply strategic solutions and choices based on the maintenance of the best possible relationship between manufacturer and retailer. Secondly, this research suggests that the manufacturer and retailer adopting a cooperative approach is, in most circumstances, the most effective route to mitigate all the challenges arising from all the aforementioned variables. Finally, this single literature review amasses most of the useful solutions suggested by scholars to managers of manufacturing companies who are engaged in the choice of strategies to use in order to optimize their on-line channel sales. In particular, cooperative advertising, monetary incentives, and cooperation in the purchasing process such as the "buy online and pick up in store approach" are shown to be valuable strategies to alleviate the manufacturer-retailer conflict in several respects.

## Research limitations

This study does have some limitations. First, the selection of papers may have been biased by the subjectivity characterizing the interpretations and evaluations of the authors. We partially addressed this aspect by implementing a multiple human subject reading and screening of the papers (Tranfield et al. 2003). The fact that Krippendorf’s alpha coefficient was an approximate 0.80 supports the robustness of our selection protocol. The second limitation is that the Scopus database was used only to perform the search phase of this research. However, we cross-validated our findings on another prestigious scientific database, Web of Science, without finding any new relevant documents.

Third, this research, amongst many others, highlights the possible conflicting relationship between retailer and manufacturer, but focuses exclusively on the manufacturer’s point of view without delving into the retailer’s perspective, although often the best solutions for both coincide. We hope our research could stimulate further investigations addressing this gap.

Fourth, our review focuses predominantly on the business and management aspects of the online channel and neglects all issues relating to the technical side of pure information systems. As an example, there is an open debate between Information Technology scholars about whether to opt for SaaS or open source CMS technologies for e-commerce platforms (Badotra and Sundas 2021; Besson and Rowe 2012). There is thus still room for future literature reviews to address the online channel management topic from different angles and perspectives.

### Future lines of research

This study finally addressed the need for an all-encompassing analysis of the emerging and fragmented literature on online channel management from the manufacturer’s perspective. The bibliographic coupling methodology facilitated the identification and subsequent analysis of three different literature clusters divided into twelve sub-clusters. From the analysis and discussion of each individual sub-cluster, an analogous number of literature gaps emerged (illustrated in Table 3), which led the authors to draft twelve research propositions to guide future research. Finally, a further theoretical gap that transcends the different sub-clusters arose from the discussion, leading the authors to formulate a final research proposition. We conclude this study by listing all the propositions formulated for the next research agenda in Table 4; this will facilitate its reading for scholars who will hopefully orient their further research on the basis of this literature review.

**Table 4:** Propositions for future research

Green cluster: strategic marketing issues within manufacturer vs retailer conflict	Blue cluster: pricing policies and price vs services trade-off	Red cluster: operational interactions and strategies between supply chain members
*P1: Researchers should expand current understanding of the market structure dynamics concerning a multiple manufacturer and multiple retailer context, basing their investigation on more realistic models or on primary data*	*P5: Future research into manufacturers’ pricing policies in the online channel could consider complex scenarios where multiple retailers but especially multiple competitors are taken into account*	*P8: Future research could focus on costs and related strategic decisions, particularly considering scenarios in which the retailer is the Stackelberg leader since in several cases they are the one with the most bargaining power*
*P2: Future research could seek to better understand if, how, and at what cost the market intelligence tools provided by external providers influence the manufacturer-retailer relationship in managing the online channel*	*P6: In the future, researchers should address the phenomenon of consumers’ bidirectional free riding between the retail channel and the direct online channel to ascertain if and under what circumstances it can affect manufacturer-retailers’ cooperation strategies*	*P9: Further research is needed to explore the spillover effect in complex scenarios, including with multiple retailers or multiple manufacturers*
*P3: Future studies focusing on the effects of manufacturer-retailer cooperation in advertising as a driver for maximising online channel profits could focus on the effectiveness of different existing practices*	*P7: Future research should consider competitive scenarios and cost acceptance due to the return rate and the eventual possibility of recycling products*	*P10: Future research should therefore consider newer supply chain structures such as ROPS and reverse showrooming, as well as how new technologies could favour new business model adaptations*
*P4: Researchers could invest in understanding how manufacturers can find ways to overcome the barriers that prevent them from profit-sharing with retailers, and whether alternative monetary incentives can mitigate these barriers or supplant this attempt outright*		*P11: Researchers should investigate the types of sales formats or digital platforms that are most suitable for the different types of strategic affordances related to the online channel beyond the profit motive*
		*P12: A further avenue for research could be to assess the impact and presumed convenience of the fulfilment partnership options from the point of view of manufacturers and market impact*
Proposition for theoretical advancements transcending thematic clusters		
* P13: The next path for the literature to follow would be to centre in on the theoretical framing of e-commerce dynamics from manufacturers’ perspective and then eventually attempt to test their viability. The Resource Based View appears to be a valid proposal, although it is not necessarily the only one to consider*		

## Data Availability

Our manuscript has no associated data.
